# Infections associated with SARS-CoV-2 exploited via nanoformulated photodynamic therapy

**DOI:** 10.5599/admet.1883

**Published:** 2023-07-01

**Authors:** Pragya Pallavi, Karthick Harini, Noureddine Elboughdiri, Pemula Gowtham, Koyeli Girigoswami, Agnishwar Girigoswami

**Affiliations:** 1 Medical Bionanotechnology, Faculty of Allied Health Sciences, Chettinad Hospital and Research Institute (CHRI), Chettinad Academy of Research and Education (CARE), Kelambakkam, Chennai, TN-603103, India; 2 Chemical Engineering Department, College of Engineering, University of Ha'il, P.O. Box 2440, Ha'il 81441, Saudi Arabia; 3 Chemical Engineering Process Department, National School of Engineers Gabes, University of Gabes, Gabes 6029, Tunisia

**Keywords:** PDT, COVID-19, photosensitizers, vaccines, drug delivery

## Abstract

**Background and purpose:**

The pandemic of COVID-19 has highlighted the need for managing infectious diseases, which spreads by airborne transmission leading to serious health, social, and economic issues. SARS-CoV-2 is an enveloped virus with a 60–140 nm diameter and particle-like features, which majorly accounts for this disease. Expanding diagnostic capabilities, developing safe vaccinations with long-lasting immunity, and formulating effective medications are the strategies to be investigated.

**Experimental approach:**

For the literature search, electronic databases such as Scopus, Google Scholar, MEDLINE, Embase, PubMed, and Web of Science were used as the source. Search terms like 'Nano-mediated PDT,' 'PDT for SARS-CoV-2', and 'Nanotechnology in treatment for SARS-CoV-2' were used. Out of 275 initially selected articles, 198 were chosen after the abstract screening. During the full-text screening, 80 papers were excluded, and 18 were eliminated during data extraction. Preference was given to articles published from 2018 onwards, but a few older references were cited for their valuable information.

**Key results:**

Synthetic nanoparticles (NPs) have a close structural resemblance to viruses and interact greatly with their proteins due to their similarities in the configurations. NPs had previously been reported to be effective against a variety of viruses. In this way, with nanoparticles, photodynamic therapy (PDT) can be a viable alternative to antibiotics in fighting against microbial infections. The protocol of PDT includes the activation of photosensitizers using specific light to destroy microorganisms in the presence of oxygen, treating several respiratory diseases.

**Conclusion:**

The use of PDT in treating COVID-19 requires intensive investigations, which has been reviewed in this manuscript, including a computational approach to formulating effective photosensitizers.

## Introduction

SARS is an airborne viral infection that spreads through mild droplets of saliva, much like the influenza virus and the common cold [1,2]. The outbreak of COVID-19 (Coronavirus disease-2019) has transformed into a global pandemic [3]. The name of the virus causing COVID-19 is given by The International Committee on Taxonomy of Viruses (ICTV) [4,5]. This became the first novel virus to emerge in the twenty-first century as both a severe and easily transmissible infection. The SARS-CoV-2 (severe acute respiratory syndrome coronavirus 2) virus is a single-stranded RNA virus that contains 29,903 nucleotides with a positive sense [6,7]. The transmission of these viruses was within animals until the end of 2019. As the seafood market in Wuhan (China), known for selling live animals like frogs, bats, birds, and snakes, has sold a virally infected organism, the infection of COVID-19 was transmitted to humans [8]. Several terrible disasters occurred throughout this phase. China's National Health Commission has provided information about the pandemic, which has been linked to viral pneumonia. Eventually, a new coronavirus (SARS-CoV- 2) was discovered following on study based on affected individuals and genetic sequence research. Furthermore, with the discovery of genetic similarities between a new coronavirus and SARS-like bat viruses, researchers hypothesized that bats might be the sole reservoirs [9-11].

**Figure 1.**
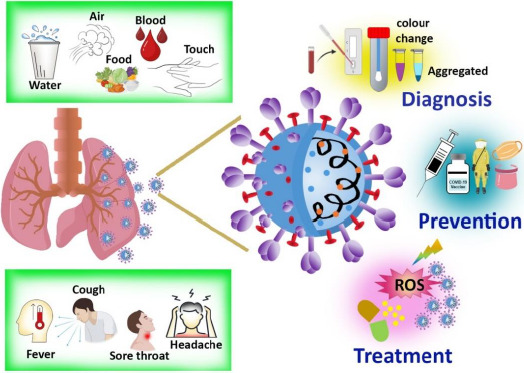
Overview of the infection and theranostic approach against COVID-19.

In order to design preventive tactics to fight an infection, the origin and transmission channels must be recognized. The majorities of infected people who are identified with COVID-19 begin the viral process three days before the beginning of the symptoms and can last up to a week after the onset of symptoms. Incubation time varies from 2-14 days in individuals who become symptomatic [12]. The overall symptoms, means of communication, diagnosis, and therapy, including prevention, are represented in Figure 1.

Fever or chills, cough, sore throat, nausea, rhinorrhea, diarrhea, weariness, exhaustion, headache, dyspnea, myalgia, dysgeusia, or anosmia are the prevalent symptoms, especially if the patients have been exposed recently [13,14]. In the majority of patients, the sickness is self-limiting, but in some cases, patients require hospitalization due to hypoxia, which is more common among those with concurrent conditions. Acute respiratory distress syndrome affects around 5 % of the population, and the infected symptomatic patient requires oxygen supplement intubation and other invasive treatment. Currently, there are many studies aiming at the development of vaccines and antiviral medicines for COVID-19. There are some antiviral drugs being prescribed for treating the COVID-19 infection, including Remdesivir, steroids, tocilizumab, favipiravir, ivermectin, lopinavir /ritonavir [15-17]. Studies revealed that in comparison to other antiviral drugs, Remdesivir showed a better therapeutic effect. A summary of the current treatment options and clinical features of COVID-19 is provided here, along with the role and progress of nanotechnology-based photodynamic therapy in treating infections.

## Methods

A comprehensive search initially yielded a total number of 275 research articles related to the topic, while some specific keywords like 'Nano-mediated PDT', 'PDT for SARS-CoV-2', and 'Nanotechnology in treatment for SARS-CoV-2' were placed in the online database search. Popular electronic databases such as Scopus, Google Scholar, MEDLINE, Embase, PubMed, and Web of Science were used to collect the articles. Instead of merely using PDT as a keyword, PDT was sometimes referred to as 'photodynamic therapy'. After conducting an abstract screening, 198 articles were selected for further consideration. During the full-text screening, 80 articles were excludeed leaving a subset of relevant articles. Additionally, 18 more papers were deemed unsuitable for inclusion in the manuscript during the data extraction process. While prioritizing articles published from 2018 onwards, a few older references were included to furnish the basic concepts utilizing their valuable and unchanging particulars.

## Vaccines

Vaccine development is a difficult, time-consuming, and expensive procedure. Trained professionals are required with multiple procedures, pauses for inspections, and data analysis to develop licensed work. The development of an ideal vaccine needs technical flexibility and adaptability with large-scale manufacturing, high purity, easy transportation, and storage. The foremost considerations while designing a vaccine are the stability, route of administration, and adverse effects. The present efforts are to create a vaccination against COVID-19 as quickly as possible. The most challenging aspect of vaccine designing is identifying the causative agent, wherein the incidence of COVID-19 was not found quickly after the outbreak began [18]. Based on the knowledge gathered from past epidemics of corona-mediated viral infections, specifically from SARS-CoV and MERS-CoV, many international funding organizations for vaccines and immunization are sponsoring to support and continue the creative attempts to design a vaccine. The protein contents of SARS-CoV-2, as well as their mode of action, had already been recognized. SARS-CoV-2 is a positive sensed and single-stranded RNA virus [19]. Proteins E, S, M, and N are some structural and functional protein that surrounds the SARS-CoV-2 virus [20,21]. The current approaches for designing vaccines constitute viral vector-based vaccines, virus-like protein particles (VLP), viral protein S subunit, and novel non-viral nucleic acid (RNA and DNA) based vaccines [22,23]. Due to the previous research in 2003 for developing a vaccine against SARS-CoV by targeting the S subunit, vaccination against SARS-CoV-2 went straight into clinical research- human trial with the previous results [24,25].

SARS-coronavirus possess unique RNA proofreading capabilities by 3'- 5' exonuclease activities of NSP12 and NSP14 [26]. This decides the two major characteristics of the virus: (1) high replication efficiency and (2) minimal new mutation rate and estimated to be around 2x10^5^. Due to the unusual faults during viral genome replication, it may sometimes result in the formation of variations, which greatly impacts the virus infectivity, causes antigenic drift, and alters the host's response towards the viral antigen. This antigen drift could hinder the construction of a vaccine that is active for a longer duration. Single amino acid mutation in the S protein, like D614G, has emerged as the widespread genotype in the present pandemic. According to preliminary studies, the D614G variation does not affect the effectiveness of vaccines targeting the S protein, as this variation has no impact on the antigenic characteristics of the S protein [27,28].

### DNA-based vaccine

DNA-based COVID-19 vaccine is a defining attempt for nucleic acid technology, mostly disregarded throughout the pandemic. A novel COVID-19 vaccine entices the immune system against the SARS-CoV-2 virus by using circular strands of DNA [29]. Researchers have hailed the clearance of DNA vaccines for humans. DNA vaccines have a number of attractive properties that include easy production with excellent quality, cost-effectiveness, high safety concerns, and stability [30]. DNA vaccines can be delivered via intramuscular or intradermal inoculation, as well as electroporation. The immunogenicity and tolerance rate of DNA vaccines were found to be huge; thus, they are being developed to combat various infections. Researchers developed a DNA vaccine in order to decrease the level of viral RNA, which was tested against a SARS-CoV-2 immunized monkey model and shown to exhibit an effective immune response [31,32]. Similarly, recombinant adenovirus expressing whole spike protein vaccine was developed and tested. Many vaccinated candidates displayed T-cell responses and dose-dependent antibody activity. The adverse effects were moderate, and no severe complications were reported [33,34].

### mRNA-based vaccine

The mRNA vaccine largely varies from conventional vaccines, where it stimulates the host immune system by using any one of two major components: an inactivated organism or an antigen, which is the protein of that organism [35]. An RNA polymerase was used to transcribe an ORF-containing mRNA from a DNA template. The advantages of mRNA-based vaccinations include simplicity, safety, and scalability of laboratory manufacturing, depending on the translational machinery of the host, and loss of integration in the genome [36]. However, there is a significant barrier to successful delivery, including the stability of mRNA during storage, the stability inside the host, an unpredictable immune response, and storage instability until frozen. Vaccination with mRNA is considered a relatively productive and time-saving option against COVID-19. As a result, RNA has emerged as the key component in the development of vaccines against COVID-19. Spike protein is the prime target for mRNA vaccines, as the structural aspect is similar to SARS-CoV and SARS-CoV-2 [37,38]. Neutralizing activity of antibodies against the N-terminal and Receptor binding domain (RBD) of S protein was studied in COVID-19 patients and proved that these are the components that hinder their use as the target for vaccine development [39,40]. The major problem is the entry route of SARS-CoV-2 into the cell with a mechanism that is not dependent on ACE2. RNA vaccine has some more challenges like the stability of mRNA, absorption efficiency by the host cells, and release rate into the cell cytoplasm to target the protein [41].

### Nanoparticle used for vaccine

RNA and DNA vaccines can generate immunity against a specific disease while reducing the risk of infection. The delivery of these immunogens to the site of action, together with other immunogens, as part of a vaccination regimen is a major difficulty. The limitations can be resolved by employing an efficient delivery method to deliver the vaccine to the target location and the adjuvant while preserving it against deterioration in a hostile environment. The delivery mechanism should have an immunogenic effect that lasts without causing any negative effects. Nanoparticles based delivery systems are popular nowadays for theranostic applications [42-45]. Nano-delivery methods might meet the criteria and allow for the long-term sustained release of vaccine molecules without being damaged by proteases [46,47]. The use of nanocarriers for vaccine components improves cellular absorption, resulting in enhanced innate, humoral, cellular, and mucosal immune responses [48,49]. Nanoparticle vaccines are powerful, safe, and simple to make. In comparison to vaccinations that retain parts of the virus, attenuated viral vaccines are more effective, but they take a longer time to reach the site, have special storage conditions (subzero temperatures), and might carry the risk of adverse effects. Vaccines containing nucleic acids (RNA and DNA) are easy to make, but they are expensive and may require many doses. There have been reports of nanoparticle vaccinations for COVID-19 eliciting an effective immunological response in mice after a single dosage. The mRNA is encapsulated in lipid-based nanoparticles or other substances and can be injected into the host via IM (Intramuscular) route to increase its stability and prevent it from rapid degradation by host ribonucleases [50]. The mRNA-1273 vaccine is the first mRNA vaccine with lipid microparticle capsule-based modified viral RNA against COVID-19 [51].

## Diagnosis

The diagnosis is made depending on a range of factors, including epidemiology, clinical findings, *in vitro* assays, and the nucleic acid amplification test. The most accurate assay for detecting SARS-CoV-2 is presently being reliably detected by real-time reverse transcriptase-PCR (rRT-PCR), which is reinforced by other auxiliary assays like serology and radiography [52]. Commercial kits have been brought into the field by validating numerous molecular and immunological aspects by the FDA (Food and Drug Administration) and ICMR (Indian Council of Medical Research) [53,54]. However, given the drawbacks in terms of sensitivity and specificity, as well as the gaps in monitoring the virus spread, there is an urgent need to develop a novel diagnostic technique that is both rapid and accurate with high safety concerns to deploy them on a wide scale in order to halt the global outbreak.

### Nanoparticle-based diagnosis

**Figure 2.**
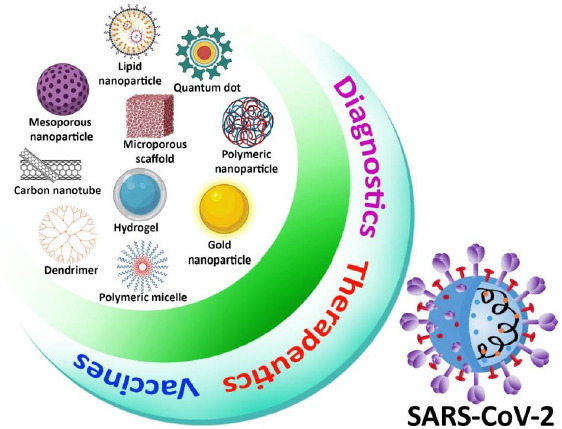
Nanoparticles used for prevention to treatment against SARS-CoV-2

Because of its simplicity, high sensitivity, and high specificity based on exponential growth in RNA generated throughout the operation, RT-PCR was used in most viral RNA detection procedures [55]. Although RT-PCR techniques are commonly accepted as the gold standard for coronavirus detection, they do have certain drawbacks, such as limited extraction efficiency, time-consuming procedure, and false-positive result due to contamination. To improve the method of detection of virus efficacy, ultra-small nanoparticles have been used not only in RT-PCR but also in other viral detection techniques such as an enzyme-linked immunosorbent assay (ELISA) and RT-LAMP (Reverse transcription loop-mediated isothermal amplification) [56,57]. Metal nanoparticles, carbon nanotubes, silica nanoparticles, quantum dots (QDs), and polymeric nanoparticles have been examined in the context of viral detection. Colorimetric, fluorescent, electrochemical, and optical imaging techniques are used as most diagnostic methods. Metal nanoparticles and QDs with distinct optical characteristics offer increased sensitivity for optical biosensing, and magnetic properties are used in the extraction process of the virus [58-61]. Nanoparticle-based viral detection method has been identified as a suitable target for SARS-CoV-2. Nanoparticles will play a major role in enhancing not only coronavirus detection efficiency but also biological pathogen diagnoses, with improvement in study and development (Figure 2) [62,63].

## Treatment

Researchers are already into the development of a variety of pharmaceuticals to combat COVID-19. Drugs being investigated for the treatment against COVID-19 follow either one of the two mechanisms: (1) to control the symptoms caused or (2) to inactivate by targeting the replication cycle of the virus [64,65]. Since no proper drug is developed, many antimicrobial drugs are being provided to the patients as a first-line treatment is tabulated below (Table 1).

## Photodynamic therapy (PDT)

**Figure 3.**
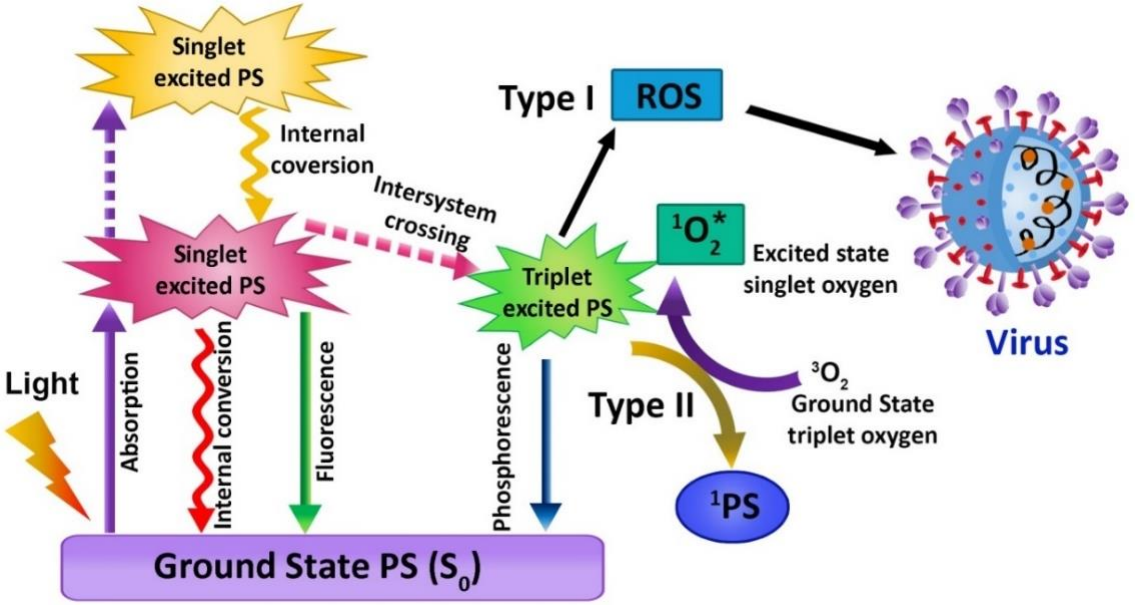
Schematic representation of the mechanism of PDT against viral infections

The use of photosensitizer (PS), light, and molecular oxygen (O_2_) has enhanced through time, and the term PDT has developed [66,67]. PDT is a non-invasive emerging therapeutic method that works by activating the photosensitizer (PS) with a certain wavelength of light. In the process of illumination, the PS transfers its energy to the molecular oxygen when it is stimulated, which in turn generates cytotoxic molecules, namely reactive oxygen species (ROS), which have the ability to disrupt the cell wall of the microorganisms in a variety of diseases and infections. The singlet oxygen produced can efficiently oxidize the main cellular macromolecules that destroy the microorganisms [67]. This therapy has been implemented to treat cancer, bacterial infection, fungal and viral diseases, as well as photodynamic diagnostics in the area of dentistry. PDT does not pose harmful risks to the biological system. It is clear that compared with traditional treatment modalities, PDT has its advantages due to its limited invasiveness and negligible cumulative toxicity. Therefore, PDT aims to boost the quality of life of patients. Jablonski's schematic diagram displays the intricacies of photodynamic activity, defined as an array of photophysical and photochemical reactions (Figure 3). PDT needs the synchronous combination of the PS, proper wavelength of visible light, and molecular oxygen. The photosensitizing molecule possesses two electrons (opposite spins) in its ground state, and the symbol S_0_ is denoted as total spin, which is zero. The reaction includes absorption of light by PS and triggers an array of photochemical reactions leading to ROS production. The ROS, also termed singlet oxygen (^1^O_2_) in the lowest energy state, can cause serious oxidative injury to pathogenic microorganisms like viruses, bacteria, fungi, and parasites. During the PDT process, it produces other ^1^O_2_, such as superoxide ions (O_2_-•), hydrogen peroxide (H_2_O_2_), and hydroxyl radicals (OH•) [68,69].

According to the mechanism of PDT, the PS excites from the low energy state to a higher energy state with a limited life cycle ranging from nanoseconds to less after the absorption of light. After excitation, any one of the two mechanisms can occur: (1) fall back to grounds state and emits fluorescence or (2) reach triplet state by undergoing the crossing in intersystem. The triplet state lifetime provides adequate time for the excited PS to interact with the oxygen molecule or other tissue substrates [70]. During this mechanism, the proton gets transferred due to this direct interaction between triplet PS and substrate, which leads to the formation of radical anion or cation, which in turn tends to react with molecular oxygen and produces oxygenated compounds like superoxide anion radical, hydrogen peroxides, and hydroxide radicals. This method is termed a type 1 reaction of PDT. In type II reaction, the direct energy transfer of the excited PS to molecular oxygen, in turn, forms singlet oxygen (^1^O_2_). Both the type I and type II reactions involve the formation of some final products, which decides the therapeutic index of the whole therapy. In a PDT reaction, both the types I and II reactions can result simultaneously [71,72]. In accordance with the category of PS, the amount of substrate in tissues, and the molecular oxygen concentration, the occurrence rate of type I and type II reaction differs. Most of the studies suggest that ^1^O_2_ plays a significant role in PDT.

### PDT for the treatment of viral infections

Infections are one of the major public health issues being studied by diverse research teams. The number of novel antimicrobials and their target structures has steadily reduced, so there is a need for alternative infection treatment [73,74]. Photodynamic therapy is considered an effective and alternative treatment option that has been shown to have great potential against a variety of disease states caused by an infectious microorganism and may be a viable option for treatment against SARS-CoV-2 infections, which can be studied further to improve the treatment success rate. The outbreak of COVID-19 has expanded enormously due to the insufficiency of immunizations also therapeutic interventions for the prevention and management of viral infections. Because of the severity and urgency of COVID-19, a new technique needs to be developed for the prevention and treatment of the virus [75,76]. Antiviral Photodynamic Therapy (aPDT) is likely to be useful as a potential therapy for coronavirus inhibition and reduction. The effectiveness of aPDT in inactivating mammalian viruses has been established in several research outcomes (Table 2).

Examples such as Adenovirus, Hepatitis viruses A, B, and C, HIV (Human immunodeficiency virus), parvovirus B19, Herpes virus, etc., causing infections have been studied to get treated with PDT, and certain studies have found that the viruses with the outer envelope are more susceptible to aPDT than non-enveloped viruses. aPDT is being utilized for inactivating multiple viruses in a variety of biological fluids, including blood. Treating superficial viral lesions with aPDT is also very effective [77]. The ROS will be produced when a photoinactivation method is performed on a variety of significant biomolecule targets, such as DNA, lipids, and proteins. ROS produces oxidative stress and causes irreparable damage to the cellular structure of the virus. This oxidative stress induces apoptosis and necrosis to kill the virus without causing damage to the surrounding healthy tissues. Both intracellular as well as extracellular ROS can be released by aPDT. PS is considered as one of the essential components of PDT. Colors of porphycenes and phenothiazines, such as azure, Methylene blue (MB), Toluidine Blue O (TBO), chlorine, porphyrin, and phthalocyanine derivatives are suitable as PS for the aPDT [78]. COVID-19 is now treated with chloroquine (C_18_H2_6_ClN_3_), and it has a structural similarity with MB (C_16_H_18_ClN_3_S). According to the preliminary data from a recent trial, MB might be an effective PS for the therapy for flu-like conditions like COVID-19. Studies proved that MB efficiently inhibits the spike protein and ACE2 (Angiotensin-Converting enzyme 2) receptor of SARS-CoV-2 to inactivate the viral replication [79].

### Nanoformulated PDT

Management of SARS-CoV-2 infections is still in progress, and some vaccines are in use in an emergency. Nanotechnology has been employed to improve the efficacy of those vaccines to target COVID-19 (Table 3). Researchers currently focus on nanomaterials-based antiviral photodynamic therapy treatment. That has been proven as an innovative strategy to inhibit bacterial and viral infections like papilloma and herpes. Nanomaterials can improve solubility, increase blood circulation time, limit enzymatic degradation, lesser unwanted side effects, and improve medication bioavailability [76]. In antimicrobial PDT, employing nanoparticles can improve the solubility, photophysics, and photochemistry of photosensitizers with targeting features.

In this process, PS can be encapsulated in different nanostructures or nanoparticles like micelles, reverse micelles, liposomes, metal and metal-oxide nanoparticles, polymer nanoparticles, carbon nanotubes, and ceramic-based nanoparticles. In this regard, the dendrimers are also promising to conjugate or load the PS molecules for better aPDT [80].

The branch-like structure and lipophilicity of dendrimers enhance the solubility of PS and, at the same time, help in the improvement of cellular uptake. The other advantages include the higher production of ROS in nanoformulations. PS molecules are well separated by the nanostructures like liposomes, dendrimers, etc., to stop self-quenching [81].

Recently up-conversion nanoparticles-based PDT has gained popularity for its absorption band in the NIR zone. After absorbing NIR radiation, the up-conversion nanoparticles (UCNP) emit visible light, which is absorbed by the PS molecules to react with molecular oxygen for the generation of ROS [82,83]. Lim et al. showed that the UCNP-mediated ROS generation could inactivate the dengue virus serotype 2 (DENV2) and adenovirus type 5 (Ad5V) [84]. This strategy can be well applicable to the management of SARS-CoV-2 infections. The surface modification strategy of nanoformulated PS with targeting moieties as specific ligands or antibodies can help to increase the specificity of aPDT against COVID-19 protecting healthy cells [85].

Carbon nanotubes have been proposed for use as novel photosensitizers in photodynamic therapy. Carbon nanotubes that have been functionalized, conjugated, or encapsulated with other photosensitizers are powerful for PDT against infectious diseases. Banerjee *et al.* investigated PpIX conjugated to multi-walled and single-walled carbon tubes, and this conjugation was found to inhibit the influenza A virus [86]. For recently discovered covid-19, surface-modified nanoparticles could target the receptor present on the lung cell with the particular antibodies causing the infected cells to be destroyed by aPDT. Recent research suggests that aPDT may be effective in the treatment of Covid-19 with lesser side effects and pharmacological interactions.

## Computational approach

The computational approach of the combination of chemistry and biology has evolved as one of the essential parts of drug discovery. New lead molecules can be significantly developed by computational approach at minimal cost within a limited period of time. The efficacy of the method of testing and analyzing the developed products can be effectively improved [87]. Certain tools like molecular docking, virtual screening, protein modeling, ADMET (adsorption, distribution, metabolism, excretion, and toxicity), molecular dynamics, and QSAR (quantitative structure-activity relationship) are capable of providing valid predictions and has raised as key part of the computational approach. The photochemical reactivity of PS needs to be well understood for an effective yield and for which molecular orbital calculations are used as they can predict the mechanism of action and anticipate the results. Wang *et al.* recently designed a PS for PDT as cancer treatment using computer-aided drug designing [88]. The team used two drugs where the similarity in molecular properties and the intermolecular interactions between the two drugs were identified with a dynamic simulation model and molecular docking, respectively. The efficiency of the particle for cancer therapy was greatly improved due to the screening test done via a computational approach [88]. Fedorov *et al*. utilized coarse graining molecular dynamic method to understand the molecular interaction between the developed PS and the surface structure of the virus, and with the same, the major and minor binding sites were also studied [89]. Similarly, Sharshov *et al*. studied the chemistry behind the interaction between PS and virus using dynamic simulation methods [90]. Table 4 summarizes the other computational approaches to formulate the PS.

## Conclusion and future perspective

COVID-19 is rapidly spreading around the world, resulting in an increase in affected cases and deaths. We are confronted with many unknowns, and we must continue to monitor and research. Until now, no appropriate therapy against SARS-CoV-2 for a complete cure is available. To resolve this issue, researchers are focusing on developing viable vaccinations and treatments. Nanotechnology is now emerging as a potential field for diagnosing and treating several disorders. Due to the small size and exponential surface chemistry, the nanoparticle can be an effective tool in manufacturing vaccines and developing treatment strategies against COVID-19 infection. PDT is a non-invasive therapeutic strategy based on the activation of a PS with the help of light in a certain wavelength. In PDT, the nanoparticle can act as a drug carrier or a drug by itself. In order to overcome the drawback of PS, nanoparticles can be used as a carrier of PS. Studies revealed nanoparticle-based antiviral medicines and nano-based-PDT would sufficiently combat the COVID-19 pandemic.

## Figures and Tables

**Table 1.** Summary of antiviral drugs being used for COVID-19 to suppress aggressiveness.

No	Name of the drugs	Class of the drug	Route of administration	Mechanism of action	Adverse effect
1.	Remdesivir	Antiviral drug	Intravenous (IV) injection	Possess inhibitory activity by inhibiting viral RNA-dependent polymerase.	Chest tightness, back pain, dark-colored urine, light-colored stools, flushing, headache, nausea, and vomiting.
2.	Dexamethasone	Corticosteroid	Oral / intravenous / intramuscular (IM) / intraarticular	Inhibit a pro-inflammatory gene encoding cell adhesion molecules (CAM).	Glaucoma, cataracts, fluid retention, hypertension, osteoporosis, mood swings, confusion, and irritation.
3.	Tocilizumab	Antiviral drug	IV infusion	Binds to both sIL-6R and mIL-6R to inhibit the action.	Stuffy nose, sore throat, headache, hypertension, and injection site reactions. The rare, more serious side effects include gastrointestinal perforations.
4.	Favipiravir	Antiviral drug	Oral	inhibit replication of influenza A and B, Ebola, and other pathogenic viral infections, but the drug has shown promise in the treatment of avian influenza.	Diarrhea, hyperuricemia, reduced neutrophil count, abdominal pain, nausea, and vomiting.
5.	Ivermectin	Antiparasitic drug	Subcutaneous / oral	It targets the IMPα component and blocks the nuclear transport of viral proteins.	Headache, dizziness, muscle pain, nausea, swollen lymph nodes, eye swelling, weakness, vision changes, and itching.
6.	Lopinavir /Ritonavir	Antiretroviral	Oral	Inhibit the action of 3CL^pro^ therapy to disrupt viral replication.	Muscle weakness, diarrhea, heart burning, difficulty in sleep, muscle pain, nausea loss of appetite
.7.	Baricitinib	Antirheumatic drug	Oral	Inhibit JAK1/JAK2 to modulate downstream inflammatory responses.	Anemia, liver disease, nausea, loss of appetite, abdominal pain, yellowing eyes/skin, dark urine, fever, fainting.
8.	Chloroquine and hydroxychloroquine	Antimalarial drug	Subcutaneous / Intramuscular	Inhibit endosomal acidification to block glycosylation and break down the production of viral proteins.	Dizziness, loss of appetite, diarrhea, blurred vision, sensitivity to light, ringing in ears, muscle weakness, and mental change.
9.	Nafamostat and comostat	Antiviral drug	Ora l/ IV	The antagonist of serine protease TMPRSS2 and inhibit SARS-CoV 2	Rash, pruritus, nausea, abdominal discomfort, and liver enzyme level elevation
10.	Fluvoxamine	Anti-inflammatory, antiviral, and antidepressant	Oral	Blocks reuptake of serotonin enhances its action	Difficult breathing, skin rash, blisters, hives, fever, joint pain, swelling of the throat
11.	Umifenovir	Antiviral	Oral	Inhibits membrane fusion of influenza virus and prevents contact between the virus and host tissues	Nausea, vomiting, diarrhea
12.	Famotidine	Histamine (H_2_) receptor antagonist	Oral	Activate the H_2_ receptor and catalyze proton pump action to secrete acid	Muscle cramps, abdominal discomfort, fatigue, anorexia, rash, dry mouth

**Table 2.** Recent findings on the effectiveness of PDT against viral infections

No	PS	Wavelength, nm	Target	Finding	Remarks
1.	5- Aminolevulinic Acid (5-ALA)	635	Juvenile laryngeal papilloma	After PDT treatment, laryngeal papilloma was almost cured, and no recurrence was observed during 6-24 months of continuous physical assessment.	PDT treatment with the combination of CO_2_ laser therapy can be helpful for the common benign tumor, which can be related to human papillomavirus (HPV) infections.
2.	Curcumin	450	Pharyngo- tonsillitis	PS was able to get delivered into the pharynx and tonsils. The microbial reduction in 1 log10 of the colony-forming unit was observed after therapy, and also recolonization was not observed after 24 h.	The use of photodynamic therapy to treat Pharyngo- tonsillitis causes only minimal side effects.
3.	Methylene blue (MB)	660	Herpes simplex virus (HSV-1)	After PDT, the first session showed betterment in anti-inflammatory activity and itching and increased the healing process of wounds.	Treating herpes simplex infections in the nasal wing with PDT and photobiomodulation was beneficial and delivering drug (PS) via nebulization can overcome drug release problems, minimizing side effects caused due to IV administration.
4.	Methylene blue (MB) and Radahlorin	662	Vero E6 cells and SARS- CoV 2	In the presence of MB and Radahlorin, the *IC*_50_ of 10^2^ TCID_50_ of SARS CoV-2 was found as 0.22 μg/ml and 0.33 μg/ml. Inhibitory concentration-50% for Radahlorin and MB was observed at 0.6 μg/ml and 2 μg/ml.	*In vitro*, when PDT is coupled with Radahlorin and MB, high antiviral efficacy against SARS-CoV-2 was observed.
5.	Phenothiazines, Methylene blue (MB), Porphyrins, Protoporhphyrine-IX (PP-IX)	-	COVID- 19	aPDT treatment using cost-effective PS could assist in mitigating COVID-19 by treating infected individuals by developing functional photoactive textiles, auto-disinfecting surfaces, and disinfecting water and air.	Photosensitizers MB and PP-IX are new technical methods to treat COVID-affected patients in a less costly and safer way by disinfecting infected water and water surface which cause the COVID disease.
6.	Methylene blue	660	Lip lesions	0.1 % methylene blue (MB) photosensitizer was applied all over lip lesions and treated with aPDT and photomodulation showed complete healing in 4 days.	COVID-19-related labial lesions can be efficiently treated by combining phototherapy modalities.
7.	Curcumin poly-lactic-co- glycolide acid	-	SARS-CoV-2, Vero cells	aPDT had anti-COVID action *in vitro* without causing Cytotoxicity in Vero cells cultured with SARS-CoV-2 treated plasma	*In vitro*, COVID-19 action was observed in treated plasma containing SARS-CoV- 2 without apoptosis in Vero cell or any negative effect on plasma quality in photodynamic therapy.
8.	5- aminolevulinic acid	635	HSIL (high-grade intraepithelial lesion)/CIN2 (cervical squamous intraepithelial neoplasia 2) with high-risk Human Papillomavirus (hHPv)	5-ALA PDT possessed higher safety and efficacy profile for the HSIL/CIN2 patients needing reproductive treatment. It can be a promising alternative surgical technique.	ALA-PDT is effective for treating cervical HSIL and high-risk HPV infections, and the effect may be maintained for a long time.
9.	Porphyrin	-	SARS-CoV-2	Coating of siloxane copolymer onto the textiles produces excitation of porphyrin that shows ^1^O_2_ oxidative crosslinking of the polysiloxane with amine links to create hydrophobic fabric.	The development of a treated mask can be a barrier and provide better inactivation of viral structures for the transmission of SARS-CoV-2 and also have broad-antiviral activity application against human-enveloped viruses such as influenza A virus and other coronaviruses.
10.	Methylene blue	-	Influenza virus, SARS-CoV- 2, and MERS (Middle East Respiratory Syndrome)	As studies suggest, MB-mediated aPDT can minimize COVID-19 impact in the absence of therapy for COVID-19.	Methylene Blue as PS for aPDT can take control of coronavirus and helps to eradicate it. More research is required for the use of antiviral PDT as a treatment against COVID-19.

**Table 3.** Nanoenabled immunization against COVID-19

No.	Vaccine		Vaccine type	Administration regimen	Target virus	Target Disease	Nanoformulation	Mode of action	Company
1.	BNT162	BNT162a1	Uridine mRNA (uRNA) vaccine	Prime/Boost (P/B) regimen	SARS-CoV-2	Covid-19	Lipid Nanoparticles (LNP)	NA	BioNTech and Pfizer
		BNT162b1	Nucleoside-modified RNA vaccine	P/B regimen				Targets the spike protein of membrane-bound SARS-CoV-2 and encodes for its prefusion state.	
		BNT162b2 or Comirnaty	Nucleoside-modified RNA vaccine	P/B regimen				Targets the spike protein of SARS-CoV-2 and encodes their receptor-binding domain, thereby eliciting human antibody and T_H_1 T cell responses	
		BNT162b3	Nucleoside-modified RNA vaccine	P/B regimen				NA	
		BNT162c2	Self-amplifying mRNA (saRNA) vaccine	P/B regimen or Single dose (SD) regimen				NA	
2.	LUNAR-COV19		Self-transcribing and replicating mRNA vaccine	P/B regimen or SD regimen	SARS-CoV-2	Covid-19	LNP	Encodes the SARS-CoV-2 spike protein and an alphavirus-based replicon.	Arcturus therapeutics
3.	Spikevax or mRNA-1273 or elasomeran		Nucleoside-modified RNA vaccine	Two dose (TD) regimen	SARS-CoV-2	Covid-19	LNP	Targets the spike glycoprotein of SARS-CoV-2 and encodes for its prefusion state.	Moderna

**Table 4.** The summary of the computer-aided designing and analysis of PS.

No	PS designed	Method used	Purpose	Ref.
1.	Palladium-Platinum complex	Gaussian 16 program	Two-photon PDT cancer therapy	[91]
2.	Imidazolium-based porous organic polymer photosensitizer	Non-adiabatic molecular dynamics simulation	Anticancer PDT	[92]
3.	Benzothiazole-based organic PS	Gaussian 16 program	Novel design of organic PS that is operative in the NIR region	[93]
4.	Halogen-free boron dipyrromethene photosensitizer	Density Functional Theory (DFT) with the dependence on time (TDDFT)	Bioimaging and anticancer PDT	[94]
5.	Propolis-benzofuran A photosensitizer	SwissADME tool, OSIRIS and Pro-Tox II	PDT for Monkeypox virus	[95]
6.	Cy7- Photolabile Protecting Groups	GaussView 6.0	Facile synthesis for biomedical applications	[96]
7.	*Meso*-tetraphenylporphyrin	TDDFT	Anticancer PDT	[97]
8.	Dicyanomethylene-4H-chromene based PS	Mesra software with Gaussian 16 program	Image-guided anticancer PDT	[98]
9.	Fucoxanthin/graphene complex	DFT	To study the photosensitization features for various applications	[99]
10.	Porphyrin-based PS with two ethanethioate	DFT and TDDFT	Calculation of the excited state properties of the novel design	[100]
